# Supplementary material for: Are carbon emissions trading and green financial instruments synergistic? -Comprehensive quantitative research based on content analysis
Source: PLoS One. 2024 Mar 7;19(3):e0298601. doi: 10.1371/journal.pone.0298601 (PMC10919601; doi:10.1371/journal.pone.0298601)
Supplement: S1 Data — (ZIP) [file pone.0298601.s001.zip › Data/地级市Z_F工作报告.zip!/┴╔─■/╜⌡╓▌╩╨/└┤╫╘╣1⁄2╓┌║┼í╛┬φ┐╦╩2╛▌═°í┐_2018.docx]

各位代表：

　　现在，我代表市政府向大会报告工作，请予审议，并请市政协各位委员提出意见。

　　一、过去五年简要回顾和2017年工作情况

　　过去的五年，是锦州发展进程中不平凡的五年。面对错综复杂的宏观环境，面对前所未有的经济下行压力，在省委、省政府和市委的坚强领导下，我们顶住压力、迎难而上，主动适应经济发展新常态，坚持稳中求进工作总基调，紧紧团结依靠全市人民，开拓创新，砥砺前行，取得了来之不易的发展成果，开创了锦州振兴发展新局面。

　　五年来，我们坚定不移推进经济转型升级，综合实力跃上新台阶。累计完成一般公共预算收入524.5亿元，比上个五年增加 125.2亿元，增长31.3%。累计完成固定资产投资3443亿元，新上10亿元以上项目103个。经济数据全面夯实，初步核算2017年实现地区生产总值1128.8亿元，位居全省第6位，三次产业比例调整为17.6:35.2:47.2，经济总量与质量稳步提升。现代农业加快发展，种植结构不断优化，调减玉米面积60万亩，粮食产量连续5年稳定在50亿斤左右，标准化养殖率达到70%，重大动植物疫情零发生，蔬菜、水果、肉蛋奶产量位居全省前列，土地流转率达到38%。实施工业强市五项工程，光伏及电子信息、石化及精细化工、汽车及零部件、生物医药等新兴产业产值以年均两位数的速度高速增长，万得汽车电器产业园、奥鸿药业、天龙化纤等一批重大项目建成投产。现代服务业蓬勃发展，滨海国家电子商务示范基地获评“全国十佳电子商务园区”，兴隆大家庭、红星美凯龙、家乐汇等一批商业综合体开业运营。国家全域旅游示范区创建全面展开，4A级景区达到12家。辽西区域金融中心建设步伐加快，上市公司和新三板挂牌企业达到15家。

　　五年来，我们坚定不移推进改革开放创新，发展动能实现新迸发。深入推进供给侧结构性改革，去产能任务全面完成，商品房去库存工作全省领先。大刀阔斧推进政府机构改革和行政审批制度改革，设立机关作风监督局，在全省率先建立督考合一机制，组建市县两级行政审批局，取消调整下放行政审批事项362项，削减率达到52.3%，审批效率和服务质量显著提升。创新两个先导区体制机制，建立大部制改革、扁平化管理、企业化服务新模式。持续推进产业园区体制机制改革，19个园区整合为9个，人员编制压缩30%。新组建8大国有企业集团，国有资产管理体制更加完善。创新驱动成果丰硕，松山高新区晋升国家高新区，获批国家产城融合示范区，国家科技成果转化服务示范基地落户锦州，高新技术企业增至94家，成立院士工作站6个，发明专利申请量年均增长32.1%。开放型经济水平全面提升，累计实际利用外资25.4亿美元、出口520亿元，锦州港口岸扩大开放获国务院批准。

　　五年来，我们坚定不移推进城乡环境建设，城市面貌发生新变化。锦凌水库建成蓄水，锦州湾机场顺利通航，“两河”治理城区段全部竣工。阜盘高速、锦赤铁路、机场公路、解放东路、市府西路、滨河路、中央南街立交桥、广州街公铁立交桥、士英街桥洞扩孔改造等一批重大基础设施建成使用。投入33.6亿元增加各级公路里程1664公里。大学城入驻师生近3万人，展现滨海新形象。东湖公园、南山公园建成使用。强力推进市容环境、农村环境、拆除违章建筑、取缔人力三轮车等10余个专项整治行动，持续开展碧水、青山、蓝天、沃土工程，锦州的天更蓝、山更绿、水更清，城乡环境更加整洁优美，城乡秩序更加文明有序，锦绣之州更加和谐宜居。

　　五年来，我们坚定不移推进民生事业发展，人民生活水平有了新提高。坚持雪中送炭、为民造福，持续不懈开展系列整治和建设年活动，财政支出用于民生的比重达到80%。全力以赴脱贫攻坚，13.6万建档立卡贫困人口稳定脱贫。7个县（市）区通过“义教均衡”国家认定，完成346所农村薄弱学校改造，尽力让每个孩子都能享有公平而有质量的教育。建成一批医疗综合楼，重大疾病补偿制度全面实施，覆盖城乡的医疗服务体系基本建立，“看病难、看病贵”问题得到有效缓解。深入实施文化建设年六项工程，精品剧目屡获大奖，群众性文化活动丰富多彩。滨海体育中心建成使用，全民健身广泛开展。牢牢抓住就业这个最大的民生，累计实名制就业24.4万人，登记失业率控制在4.26%以内，新建人力资源市场投入使用。社会救助标准持续提高，城乡居民养老保险实现全覆盖，建成市养老综合服务中心，社会保障体系更加完善。累计完成棚户区改造27875户，保障性住房建设稳步推进。新增新能源公交车486辆，市民出行更加绿色便捷。荣获全国双拥模范城“四连冠”。食品药品安全、群众信访、安全生产等工作不断加强，刑事发案率下降32.9%，锦州大局保持和谐稳定。

　　五年来的发展成就令人振奋，五年来的发展历程充满艰辛。特别是2014年国家实施新一轮东北振兴战略以来，市委统揽全局、协调各方，人大、政协鼎力支持、有效监督，全市人民埋头苦干、奋力拼搏，推进了一系列重大举措和重点工作落地落实，解决了许多长期想解决而没有解决的难题，办成了许多过去想办而没有办成的大事。这些重大成就与巨大变化，必将对锦州决胜全面建成小康社会产生重要而深远的影响。

　　各位代表！2017年是本届政府任期的最后一年，按照新发展理念和“四个着力”“三个推进”要求，全市人民凝心聚力，克难奋进，较好完成了年初确定的目标任务。

　　初步核算，全市地区生产总值增长6.2%，规模以上工业增加值增长4.4%，一般公共预算收入91.2亿元、增长7.6%，固定资产投资357.8亿元、下降5.8%，社会消费品零售总额634.6亿元、增长3.9%，城镇和农村常住居民人均可支配收入分别达到30412元和14493元、分别增长6.8%和7%，居民消费价格涨幅1.4%。经济实现企稳回升，主要经济指标增幅高于全省平均水平。

　　(一)深化改革力度空前

　　以“三去一降一补”为重点，深入推进供给侧结构性改革。关闭9万吨/年以下煤矿16处，化解产能89万吨。商品住房去化周期降至9.4个月。清理废止涉企收费文件604份，建立收费目录清单。22户企业参与直购电交易，节约用电成本7000余万元。加大力度补短板，投入扶贫专项资金 1.3亿元，发放扶贫贷款8.2亿元。

　　持续推进“放管服”改革，“双随机、一公开”监管实现全覆盖。整合成立市公共资源交易中心，公共资源配置质量和效益明显提高。

　　开展优化营商环境建设年活动，出重拳实施系列专项整治，查处一批破坏软环境典型问题，企业和群众满意度不断提高。

　　学习上海、天津等自贸区经验，强力推进先导区体制机制创新，滨海、松山两区工管委工作机构分别由32个减至20个、29个减至17个。

　　深化国企改革，热力、水务等8大国有企业集团共整合企事业单位68家，净资产达到78亿元。

　　实施医改攻坚，扎实推进国家第四批公立医院综合改革试点。公立医院全部实现网上药品采购和零差率销售。创建分级诊疗新模式，组建7个医联体。初步建立“一局一中心”医疗保障管理体制。

　　国有林场改革、事业单位改革、供销社综合改革等取得新进展。

　　(二)项目建设持续发力

　　制定出台一系列实施方案和行动措施，全面对接新一轮振兴东北政策，争取国家和省项目计划支持。汉拿驱动电机项目列入国家工业强基工程计划，德商新能源汽车等33个项目列入省重大工业项目三年滚动计划，辽晶电子等10个项目列入省“四基”工程计划。

　　开展招商引资项目建设年活动。积极“走出去、请进来”，成功举办锦州籍、江苏和京津冀企业家锦州行等系列招商活动。全年新引进总投资亿元以上项目78个，引进省外资金到位额200亿元，实际利用外资1.3亿美元。1000万吨/年氧化铝、200万吨/年乙烷裂解制乙烯、25万吨/年聚酯和20万吨/年FDY纺丝装置等具有重大影响的项目前期工作稳步推进。

　　全市在建亿元以上项目208个，完成投资269.3亿元。沃特玛新能源汽车创新产业园、大北农生猪繁育及加工等10亿元以上项目开工建设。凌海光和史迪威甜菊糖、义县九丰肉鸡屠宰深加工等项目竣工投产。

　　与江苏对口合作推进项目40余个，与苏州建立对口合作机制。继盘锦之后，又同葫芦岛签订全面交流合作框架协议。

　　锦州港完成货物吞吐量1.07亿吨、集装箱运输123万标箱。锦州-俄罗斯中欧海铁班列首次开通。国际贸易“单一窗口”正式运行。锦州湾机场旅客吞吐量突破20万人次，增开锦州-成都航线。

　　大有经济区和汤河子产业区晋升为省级经济开发区。

　　（三）结构调整有力有效

　　加快工业转型升级。锦懋光伏500MW太阳能组件、德瑞克汽车配件、天工半导体大尺寸硅片、神工半导体三期等一批特色新兴产业项目开工建设或投产达效。锦州钛业等3家企业获首届市长质量奖。落实市级领导帮扶重点企业制度，组织产销对接会，开展金融进园区活动，工业经济逐步趋稳向好。召开全市促进民营经济发展大会，表彰100户企业、50名企业家，出台10项扶持政策。获批省智能制造及智能服务试点示范标杆企业1家、示范项目3个。新增省“专精特新”产品（技术）13个。

　　推进农业提质增效。新建设施农业小区16个，节水灌溉12.9万亩，新增“三品”认证60万亩，主要农作物综合机械化率达到86%。抗旱、秸秆综合利用、病死动物无害化处理等工作取得显著成效。锦州苹果地理标志证明商标成功注册，北镇葡萄获国家级特优区认证，黑山褐壳鸡蛋获批创建国家级农产品地理标志示范样板。完成农村土地承包确权登记颁证708万亩。新增家庭农场200个。农产品质量安全追溯体系逐步健全。

　　现代服务业快速发展。万达广场、爱一方等商业综合体开业运营，古塔太阳广场购物中心、凌河百脑汇资讯广场等即将开业，凌河SK农副产品国际物流园、太和辽西建材物流中心等项目加快建设，富民农业等5户企业获评省农产品冷链物流区域示范企业，辽西小商品等3个市场入选省重点商品交易市场，京东商城东北地区首个城市仓落户锦州。71家企业依托阿里巴巴平台开通B2B电商业务，乡村电商服务站覆盖率达到80%。黑山村淘成为全国首批东北唯一农村淘宝创业孵化中心。锦州世博园和北镇大朝阳温泉山城晋升国家4A级旅游景区，辽沈战役纪念馆入选首批“全国中小学生研学实践教育基地”，义县碾盘沟攀岩基地获评中国体育旅游精品景区。全年实现旅游收入195亿元，增长12%。辽阳银行、营口银行落户锦州，捷通铁路在新三板挂牌。

　　(四)创新发展动力强劲

　　制定科技创新发展三年行动计划，实施八项行动。出台12项人才引进扶持政策。成功举办科技成果转化对接等系列活动。大学科技园建成开园，辽宁汇普园生物医药等14个项目签约入驻。锦州银行科技金融服务中心挂牌运营。

　　国家级监督检验中心通过验收，组建省级重点实验室1个、工程技术研究中心4家、产业专业技术创新平台6个、产业技术创新战略联盟4个，促成中科院院地合作项目8个。争取省产业（创业）投资引导基金直接投资7000万元。8项成果获省科技进步奖。新增众创空间4个、省级知识产权优势企业3家。

　　出台智慧城市建设总体方案。华为云平台上线运营，华为锦州公安云样板点建设启动，必爱旅行网等4家企业与高新区签订战略合作协议。启动建设政府协同办公系统。建成数字化城市管理平台。

　　(五)城乡建设成效显著

　　完成徐州街改造、滨河路三期、锦铁步行桥改建等工程，城市道路通达性进一步提升。“两河两岸、两路一街”亮化工程使夜色中的锦城更加靓丽。滨海新区、锦州南站两个公路客运枢纽主体完工，南站广场整体改造竣工投入使用，城市功能进一步完善。锦州绕城公路一期、广州街跨小凌河大桥、中环西路续建等重大工程已完成开工前准备工作，即将开工，锦城人民多年的企盼正在变为现实。

　　全市一盘棋、全民总动员，全面开展创建国家卫生城市工作。北镇市创建国家卫生城市通过省评估。

　　着力打通城市“微循环”，下功夫解决群众身边事。改造建设城区小街小巷和县乡村路1.4万条，修砌道路边沟130万延长米，安装路灯2.2万基，人民生活环境显著改善，城乡群众出行更加便捷。加快南山垃圾中转站建设，维修改造城市供热管网，科学施划交通标志标线，强化静态车辆管理，“城市病”正在得到有效治理。

　　集中开展市容环境综合整治“双百”行动。坚持边整治边建设边修复，投入5亿元突出整治城市脏污和乱象，完善城市功能。修补破损路面4.6万平方米，新栽补植花草树木63万株，建成休憩角(亭)125个。拆除违章建筑1758处，特别是拆除了多年想拆除而未拆除的建筑物8处，解决了多年想解决而未解决的“肠梗阻”问题，一批影响城市形象的“顽疾”得到有效治理。主要街路、弃管小区、露天市场等重点区域面貌焕然一新，“双百”行动得到了广大市民的高度赞誉。

　　圆满完成宜居乡村建设三年行动目标，实现宜居达标村全覆盖。所有行政村主街主路全部硬化，文化广场、应急广播等便民服务设施全部建成，卫生保洁制度全部建立，农村人居环境全面改观。北镇沟帮子获批国家级特色小镇。

　　(六)生态环境大幅改善

　　全面实施环境保护和建设三年行动计划。中央环保督察组交办环境信访案件全部办结。完成生态红线划定工作。

　　拆除燃煤小锅炉212台，淘汰黄标车及老旧车辆8109辆，秸秆焚烧得到有效遏制，空气质量不断改善。

　　小凌河城区段河道防渗工程、女儿河南岸生态修复工程如期完工，小凌河成功蓄水，锦城呈现城水相依、水清岸绿美丽风景。

　　人工造林10.6万亩，封山育林8.1万亩，治理水土流失32.8万亩。创建省级生态乡镇2个、生态村16个。锦州国家地质公园建设通过国土部验收。

　　海岸带生态建设三年行动计划初见成效。“蓝色海湾”整治修复项目稳步推进，取缔“三无”渔船293艘。大笔架山、大凌河口两个国家级海洋公园双双获批。

　　(七)民生事业成果丰硕

　　实施产业扶贫项目419个，24537人脱贫、61个贫困村销号，超额完成省定脱贫攻坚任务。义县脱贫攻坚工作得到省委、省政府充分肯定。

　　社会保障更加有力。出台6项吸引高校毕业生来锦工作政策。城镇新增就业1.7万人，零就业家庭保持动态为零。继续上调企业退休人员基本养老金和城乡低保标准。148家养老机构与医疗卫生机构签订服务协议。实施1200户贫困残疾人家庭无障碍改造。

　　全面完成基础教育建设年目标任务。新改扩建公办幼儿园17所，普惠性幼儿园学位覆盖率达到68%。完成锦州中学整体搬迁，办学条件彻底改善。“三通两平台”建成投入使用，教育信息化水平大幅提升。

　　文化建设年取得新成果。成功举办锦州籍书画名家作品展和辽西五市民俗文化节等大型活动。评剧《凌河村》获省艺术节“文华奖”金奖。电影《为了这片土地》、歌曲《我的国家》获省“五个一工程”优秀作品奖。锦州文化艺术中心主体工程完工。

　　成功举办全国少年田径锦标赛、市首届农民运动会等多项赛事，我市健儿在全运会获2金1铜好成绩。

　　实施食品药品安全整治三年行动计划。开展系列专项整治行动，加强分级监管，食品药品安全得到有效保障。

　　平安锦州建设成效显著。派出所基础建设三年规划启动实施，人民群众安全感、满意度全省领先。推进“七五”普法，基层人民调解覆盖面达100%。开展清仓起底攻坚行动，一大批信访积案得到有效化解。严格落实企业安全生产主体责任，安全生产形势保持稳定。应急处置、防灾减灾能力进一步提升。

　　(八)自身建设全面加强

　　党的建设持续加强。市政府党组以上率下，认真学习贯彻党的十九大精神，推进“两学一做”学习教育常态化制度化。深刻汲取辽宁拉票贿选案教训，严守党的政治纪律和政治规矩，坚定自觉地在思想上政治上行动上同以习近平同志为核心的党中央保持高度一致，确保中央和省委、省政府及市委各项决策部署不折不扣落到实处。

　　法治政府建设持续加强。严格政府立法程序，规范行政执法行为，依法行政工作全省领先。推进政务公开，坚持重大事项向社会公示或听证。主动接受人大法律监督、工作监督和政协民主监督，认真听取各民主党派、工商联、无党派人士和人民团体意见。76件人大代表建议、225件政协提案全部办复。

　　作风建设持续加强。出台加强政府系统廉政建设实施意见。强化督考合一和审计监督，严肃整治不作为乱作为行为。严格执行《准则》《条例》和中央八项规定精神，严厉惩治各种顶风违纪行为。政府系统风清气正、干事创业氛围日益浓厚。

　　国家安全、人防、气象、地震和民兵预备役等工作不断加强，民族宗教、外事、台务、慈善、档案、地方志和住房公积金管理等工作取得新成果。

　　各位代表！回顾五年来的政府工作，锦州发生了令人鼓舞的巨大变化，这是省委、省政府和市委正确领导的结果，是全市人民戮力同心、奋力拼搏的结果，也得益于历届市委、市政府打下的良好基础。在此，我代表市政府，向在各个领域和岗位辛勤劳动、做出贡献的全市人民，表示崇高的敬意！向给予政府工作大力支持的人大代表、政协委员、各民主党派、工商联和各人民团体，向关心和支持政府工作的离退休老同志，向驻锦中省直单位和解放军指战员、武警官兵，向所有关心支持锦州振兴发展的海内外朋友，表示衷心的感谢！

　　在看到成绩的同时，我们也清醒地认识到，发展中还存在不少困难和问题：一是经济总量偏小，缺少具有重大支撑作用的企业和项目；二是区域发展不平衡，创新活力不足，县域经济增长乏力；三是沿海区位和港口优势还未得到充分发挥，临港产业亟待做大做强，陆海统筹、港城联动还需强力推进；四是财政收支矛盾突出，民生保障压力较大，防风险任务还很艰巨；五是个别干部懒政怠政、推诿扯皮，群众办事难等问题仍然存在，个别领域腐败问题时有发生，软环境建设还需进一步加强。我们一定要直面问题，敢于担当，全力以赴做好政府工作，不辱历史使命，不负全市人民重托。

　　二、今后五年工作展望和2018年工作安排

　　今后五年，是“两个一百年”奋斗目标的历史交汇期，是全面建成小康社会的决胜期，更是锦州建设辽西区域中心城市的攻坚期。省委、省政府对锦州振兴寄予厚望，全市人民对锦州发展充满期待。任务艰巨，时不我待。

　　我们要不忘初心、牢记使命，准确把握锦州后发优势迸发、多重政策叠加、动能转换积蓄的阶段性新特征，深入学习贯彻党的十九大精神，以习近平新时代中国特色社会主义思想为统领，按照新发展理念和“四个着力”“三个推进”要求，认真落实省委、省政府和市委的决策部署，抢抓新一轮东北振兴机遇，坚持稳中求进工作总基调，以深化供给侧结构性改革为主线，加快建设现代化经济体系，持续保障和改善民生，推动锦州振兴发展，坚决打赢新时代“辽沈战役”锦州攻坚战。

　　全市经济社会发展目标是：经过三年努力，到2020年末，力争实现地区生产总值1400亿元、一般公共预算收入120亿元，与全国全省同步全面建成小康社会，初步形成辽西区域创新研发中心、新兴产业和先进制造业中心、科教文化和医疗卫生中心、金融服务中心、商贸物流中心、信息数据中心和海空航运中心，城市综合竞争力显著增强。再经过两年奋斗，到2022年末，全市经济实力实现大幅跃升，战略性新兴产业、骨干企业支撑作用更加有力，产业产品结构更加合理，商贸金融集聚度更加丰厚，法治体系和多层次社会保障制度更加完善，生态环境更加优美，各类要素更加齐备，辽西区域中心城市框架基本形成，迈进全省振兴发展前列。

　　围绕上述目标，我们要深入实施“五大战略”，加快建设“五个锦州”：

　　——坚持发展经济、推动振兴，加快建设实力锦州。以质量第一、效益优先为原则，深入实施以港兴市、创新驱动战略，积极扩大对外开放，大力发展实体经济，以重大临港产业项目、战略性新兴产业为牵动，统筹推动沿海、县域、城区发展质量变革、效率变革、动力变革，不断增强经济创新能力和竞争实力。

　　——坚持深化改革、依法治市，加快建设法治锦州。以法治思维和法治方式不断深化供给侧结构性改革，推进体制机制、国资国企、农业农村、社会事业等各领域改革，加快法治政府、法治经济、法治社会一体化建设，为振兴发展提供体制机制保障和良好法治环境。

　　——坚持文化牵引、科教支撑，加快建设文明锦州。以创建全国文明城市为载体，大力发展文化事业和文化产业，全面提升城市文化品位，优先发展教育事业，做好人才开发工作，打造教育和人才高地，不断扩大锦州的文化感召力、科教支撑力、社会凝聚力和城市吸引力。

　　——坚持城乡统筹、绿色发展，加快建设生态锦州。加强城市规划建设管理，着力提升城市功能和宜居水平。实施乡村振兴战略，加快农业农村现代化和城镇化进程。深入实施生态立市战略，强化生态文明建设，着力解决突出环境问题，加大生态系统保护力度，走好绿色发展之路。

　　——坚持民生为本、全民共享，加快建设幸福锦州。坚持以人民为中心的发展思想，抓住人民最关心最直接最现实的利益问题，完善公共服务体系，提升公共服务供给保障能力，创新社会治理方式，打赢脱贫攻坚战，促进社会公平正义，不断满足全市人民日益增长的美好生活需要。

　　各位代表！2018年是贯彻党的十九大精神的开局之年，是新一届政府的起步之年，能否圆满完成今年各项工作任务，事关党的十九大精神在锦州落地生效，事关决胜全面建成小康社会，事关加快实现锦州全面振兴。

　　经济社会发展主要预期目标是：地区生产总值增长7%，规模以上工业增加值增长7%，一般公共预算收入增长7%，固定资产投资增长9%，社会消费品零售总额增长8%，城镇和农村常住居民人均可支配收入均增长7%。

　　确定以上目标，主要考虑今年是决胜全面建成小康社会的关键之年，要与“十三五”规划目标相衔接，与全国全省同步全面建成小康社会相适应，与省、市三年攻坚行动计划相一致。起步就要提高站位、拉高标杆，也有利于凝聚力量、提振士气。同时，宏观经济逐渐回暖，我市经济企稳回升，为我们实现上述目标提供了有利条件。在实际工作中，我们将自加压力、奋力争先，朝更高目标努力，力争主要指标增速高于全省平均水平。

　　为实现上述目标，重点抓好以下九个方面工作：

　　（一）坚持深化各项关键改革，切实增强发展内生动力

　　全面实施我市供给侧结构性改革总体工作方案和工业、农业、服务业等具体行动计划。坚持去产能、去库存、去杠杆、降成本、补短板，优化存量资源配置，扩大优质增量供给。

　　深入推进“放管服”改革。学习借鉴苏州经验，建立阳光高效审批制度，实施精准协同放权。全面清理审批事项流程要件，打造一流网上审批服务平台，启动行政审批全程代办、全流程服务，审批效率再提速10%。深化商事制度改革，提升企业注册便利化水平，切实加强事中事后监管。进一步完善公共资源交易平台建设。

　　全领域全要素优化营商环境。坚持问题导向开展专项整治，建立部门信息共享和协作机制，解决行政执法、政府诚信等领域突出问题。

　　深化国资国企改革。基本形成以管资本为主的国资监管体制，确保国有资产保值增值。推进顺达集团转型成为国有资本投资运营公司。完善国企集团法人治理结构，配齐配强董事、监事队伍。出台差异化薪酬分配办法，铺开企业内部三项制度改革。妥善处理国企改革遗留问题。

　　深化先导区体制机制创新，加快形成可复制可推广经验做法。深化产业园区管理体制改革，创新园区绩效考核体系和人事管理办法。

　　加强政府性债务管理，严格控制新增债务，有效防范化解债务风险。加强养老金收支管理，多渠道筹措资金，确保养老金按时足额发放。

　　坚持统分结合、管放结合，推进县乡财政体制改革，建立县乡财政收入增长机制，激发乡镇（街道）发展活力。

　　统筹推进政府机构、经营性事业单位、城市管理执法体制、土地制度、供销社等各项改革。

　　（二）坚持以锦州沿海经济带建设为突破，形成港产城融合发展新态势

　　依托锦州沿海区位与港口优势，积极融入“一带一路”建设，抢抓全省“五大区域发展战略”机遇，启动实施《辽宁沿海经济带锦州市发展三年攻坚计划》，加快滨海、松山两个先导区创新发展和凌海“转身向海”开放发展，构筑“2+1”两区一市海陆结合、三箭齐发、率先发展新格局，打造引领锦州振兴发展新引擎。“两区一市”主要发展指标位居全市前列。

　　做大做强锦州港。支持锦州港参与辽宁港口资源整合。实施30万吨级航道浚深项目。港口货物吞吐量、集装箱运输稳定在亿吨和百万标箱以上。深化大通关改革，推动国际贸易“单一窗口”全面运行，争取设立锦州港保税物流中心（B型）。推进辽蒙欧综合运输大通道建设，持续增加中欧海铁班列货运量。

　　推动临港产业集聚发展。全力推进1000万吨/年氧化铝、200万吨/年乙烷裂解制乙烯、25万吨/年聚酯和20万吨/年FDY纺丝装置等重大临港产业项目开工建设。加快推进沃特玛新能源汽车配套等项目建设。力争德商新能源汽车整车下线、阳光能源800MW太阳能组件等项目投产达效。

　　全面提升滨海新区城市功能。加快北大青鸟附属学校、北医三院医养结合中心等项目建设进度。推进锦州医科大学、锦州师专、辽宁理工学院、锦州医科大学医疗学院等迁建工程，壮大滨海大学城规模。

　　促进国家高新区提档升级。集聚创新要素加快大学科技园发展，打造锦州“中关村”。确保锦州北航科技园、上海复星原料药生产基地等项目开工建设，万得科技大厦等项目建成投入使用。全面推进松山国家产城融合示范区建设。

　　设立县域（凌海）沿海经济改革试验区。支持凌海市南扩西进，逐步实现转身向海、锦凌同城化。围绕“三横七纵”公路网建设构筑产业布局，加快九华山特色小镇和朝凌客专凌海南站建设，推进双羊传化公路港、国药物流、龙海馨港、东方华地城二期等项目建设，努力打造新兴工业产业集群、现代服务业集聚区、农业科技园区。

　　（三）坚持增量与存量并举，再掀招商引资和项目建设新热潮

　　全面贯彻中央7号、国务院62号等文件精神和省委、省政府决策部署，精准对接，跟踪问效，争取国家和省更多政策、项目、资金落地锦州。实施《锦州市经济振兴发展攻坚行动方案（2018-2020年）》，以“2+5+2”国家、省、市三级产业园区为载体，全力打造10大产业集群。

　　扎实开展招商引资项目建设年活动。集中力量打赢项目攻坚战。全年安排亿元以上项目210个，完成投资270亿元。引进域外资金到位额360亿元，实际利用外资3亿美元。坚持面向长三角、珠三角、京津冀地区，特别是雄安新区及对口合作的苏州市等地开展定向招商，突出产业链招商、以商招商和专业招商。坚持引资引智并重，开展“锦上添花”招商行动，加快重大项目和高端人才引进。坚持抓大活小，实施“繁花似锦”项目建设计划，全力抓落地开工、抓投产达效。努力形成大项目顶天立地、小项目铺天盖地的生动局面，为建设“锦绣之州”奠定坚实的项目基础。

　　坚持“一个项目、一名领导、一套班子、一抓到底”工作机制，建立重点项目推进承诺制，狠抓责任落实和考核问责，明确时间表、路线图、任务书，实行挂图作战、销号管理，月调度、季拉练。

　　着力促进民营经济发展。强化组织领导，全面落实10项扶持政策，提高民营经济发展集中度丰厚度。

　　优化对外贸易结构。支持滨海新区光伏、汤河子半导体硅片、七里河机电装备等产业集聚区创建外贸转型升级示范基地。实施境内外“百企百展”市场开拓计划，深度拓展国际市场。全市地方企业外贸出口增长5%。

　　全力推进与苏州在城市建设、规划引领、平台载体等方面对口合作，推动两市合作向纵深发展。加强与大连交流合作，建立长期合作机制，实现同辽宁沿海经济带核心城市互联、互补、互动发展。

　　（四）坚持着力振兴实体经济，全力建设工业强市

　　持续抓好工业强市五项工程。强化对重点行业和100户重点企业监控调度，加大规下企业培育和升规力度，帮助企业解决生产经营难题。着力培育高级应用领域钛白粉、钼酸铵、半导体材料、高端成套装备等一批市场前景广、牵动力大的新产品。推动互联网、大数据、人工智能和实体经济深度融合，提升企业两化融合水平。推进华润锦州电厂热电机组、锦州至高台山铁路扩能改造、锦州城东变等三项电力工程建设。

　　加强园区基础设施建设。盘活闲置厂房，拓展园区空间，省级及以上开发区新增建成区面积不少于1平方公里，工业园区规模以上企业数量、企业主营业务收入保持两位数增长。

　　培育壮大企业创新主体，全市高新技术企业突破100家，科技型中小企业达到60家。组建一批省级以上工程技术研究中心、重点实验室和众创空间等创新创业平台，培育引进一批领军型科技创新创业人才。积极争取省产业（创业）直投基金，支持发展知识产权质押融资风险补偿基金。促进企业与高校、科研院所合作，加速科技创新成果在锦转化。强化知识产权创造、保护、运用，提高万人发明专利拥有量。

　　（五）坚持全面实施乡村振兴战略，强力推进县域经济发展壮大

　　实施《锦州市推进县域经济发展全面建成小康社会三年行动计划》，推动新型工业化、信息化、城镇化、农业现代化同步发展。

　　按照“县扩园区、乡增活力、村壮集体”的发展思路，着力发展县域企业，强力推进“一乡一业一村一品”。各县（市）新上亿元以上项目10个以上；固定资产投资增长15%。资源条件好的乡镇（街道）新上3000万元以上项目2-3个，其他乡镇（街道）新上500万元以上项目2-3个。充分利用农村土地确权成果，对实测面积超出确权面积的土地，依法盘活用好，发展壮大村级集体经济，逐步消灭集体收入“空壳村”。

　　建立完善市级领导和市直机关包扶县域经济发展制度，加大政策倾斜力度，设立县域经济发展专项基金。强化指标考核，四县（市）主要指标增速要高于全省县域平均水平、居民人均可支配收入增幅要高于地区生产总值增幅。

　　加快推进农业现代化。新建5个高标准设施农业小区，大力培育新型农业经营和服务主体，引导农村土地适度规模经营。落实粮食安全生产责任制。加强畜禽粪污处理、动植物疫病防控，健全农产品质量和食品安全监管体制。依托龙头企业，大力推广“公司+基地+农户”模式，提高农产品深加工水平。主要农作物综合机械化率达到88%。完成承包地确权登记颁证，稳步推进农村集体产权制度改革，落实“三权”分置办法。

　　推进新型城镇化建设。打造一批具有一定产业基础、文化氛围浓厚、地域特点鲜明的产业特色小镇。开展新一轮宜居乡村建设。

　　开展好第三次全国土地调查，坚守耕地和基本农田保护红线，提高土地节约集约利用水平。做好第四次全国经济普查工作。

　　（六）坚持以发展现代服务业为重点，推动城区差异化特色化发展

　　实施《锦州市城区经济发展三年攻坚计划》。古塔区要加快建设精细化工、电子信息产业园，着力打造北普陀山文化旅游集聚区及锦凌水库生态旅游区，发展商贸流通、总部经济等产业。凌河区要积极引进国内外企业总部、研发中心、结算中心等，加快发展旅游产业和创新经济，抓好中央大街、小商品批发市场等商圈提档升级。太和区要着力发展现代物流、专业市场、生态旅游等产业，以汤河子开发区为核心大力发展新材料等战略性新兴产业，依托南庄里文创城发展壮大文化产业。

　　促进传统服务业转型升级。举办系列精品促销活动，激发消费市场活力。推进盛世二手车交易、九华山小商品批发、辽西果蔬批发等市场争创省转型升级示范商品市场。推进海尔（锦州）虚实网服务园、杏山物流园等现代物流园区加快建设。提升南郡天下、南阳路等一批特色商业街区建设标准。

　　发展创新型服务业。以国家电子商务示范基地等平台为重点，推进一批电子商务平台和网上商城建设，开展电子商务示范企业、基地创建活动。推动电子商务进社区，完善农村电子商务服务体系。积极引导企业利用跨境电商平台开展进出口业务。

　　加快建设辽西区域金融中心，壮大松山金融产业集群，大力发展产业金融，引进金融机构3家，争取新增上市公司、新三板挂牌企业3家。

　　开展旅游建设年活动，做好全域旅游示范区创建工作。创新国有景区管理体制机制。举办大型红色旅游推广活动。大力发展乡村旅游，实施环闾山游大通道建设。加快市县两级旅游集散服务中心建设。新建、改建旅游厕所155座。推进5A级景区规划创建工作。实现旅游收入218亿元，增长12%。

　　全面实施智慧城市建设总体方案。依托华为辽宁大区（锦州）云计算数据中心，推进政务信息系统整合共享和智慧交通、警务、医疗、旅游等信息化应用，加快云计算和大数据产业发展。

　　（七）坚持规划建设管理并重，着力提升城乡人居环境质量

　　加强规划引领，依法维护规划严肃性。坚持多规合一，推进新一轮城市总体规划修编。

　　全力推进锦州绕城公路一期、广州街跨小凌河大桥、中环西路续建、女儿河左岸滨河路等重点工程建设。启动102国道改线工程。实施解放西路拓宽改造和南广路拓宽及地下综合管廊建设工程。规划建设市档案馆、城市规划展示馆、城市文化公园。启动南山垃圾填埋场封场工程，推进混合垃圾填埋场建设。确保锦州南站公路客运枢纽竣工使用。加快建设天然气管网，新建CNG、LNG储气装置，提升燃气供应安全保障能力。

　　以创建国家卫生城市为引领，巩固提升城市美化绿化亮化净化成果。实施小街小巷改造70条，打通城市规划区范围内部分“断头路”，城区道路机械化清扫率达到85%。开展城市架空通讯线路整治行动。实施“厕所革命”新三年计划，新建、改建61座城区厕所。城市规划区内非物业小区逐步实现准物业管理全覆盖。全面提升城市网格化精细化管理水平。

　　以大气污染防治为重点，深入实施环境保护和建设三年行动计划。加快建成区供暖并网改造，拆除燃煤小锅炉52台。确保工业园区10吨以下燃煤锅炉全部拆除。实现全域秸秆禁烧，提高秸秆综合利用率。加大水源地保护力度，提升污水处理能力，确保域内河流断面达标。开展土壤污染状况调查，落实土壤污染防治方案。

　　继续创建省级生态县、乡、村。完成全市河长配置工作。实施农村饮水安全巩固提升工程。人工造林6.9万亩，封山育林2.5万亩，完成森林防火综合治理项目。

　　深入实施海岸带生态建设三年行动计划。统筹推进两个国家级海洋公园建设。“蓝色海湾”整治修复项目通过国家验收。严格依法用海管海，加快近海渔业生态恢复。

　　（八）坚持保障和改善民生，不断提高人民群众幸福指数

　　始终坚持以人民为中心的发展思想，全面落实省城乡居民和各类群体收入增长三年行动计划，不断提升城乡居民收入水平。

　　把脱贫攻坚作为头号民生工程来抓，产业扶贫、行业扶贫、社会扶贫综合发力，确保8300人脱贫、56个贫困村销号。

　　提高就业质量。大力开展就业援助，多渠道促进高校毕业生就业。城镇新增就业1.5万人，登记失业率控制在5%以内。

　　加强社会保障体系建设。推进机关事业单位养老保险制度改革。城镇职工基本养老保险人数达到77.8万人。继续提高最低生活保障和特困供养标准。启动养老事业发展三年行动计划。推进保障性安居工程建设，改造棚户区5600户。搭建租售服务平台，发展壮大住房租赁市场。实施贫困残疾人家庭无障碍改造1000户。

　　优先发展教育事业。制定教育发展中期规划，实施基础教育质量全面提升工程、普通高中质量提升行动计划。开展学前教育建设年活动，研究制定三年发展规划。义县和松山新区通过“义教均衡”国家评估认定。普惠性幼儿园学位覆盖率达到72%。大力发展职业教育，促进产教融合、校企合作。推进锦州中学、机电工程学校建设辽宁名校。

　　切实抓好公立医院综合改革。加快建立现代医院管理制度，完善分级诊疗，开展紧密型医联体试点。扎实做好“三医联动”改革试点工作，推进医保支付方式改革和管理机构整合。启动基层卫生建设年活动，基层医疗机构人员编制、经费全部上划县级管理，上级医院医务人员轮岗下派到基层医疗单位工作。市中心医院门诊楼投入使用，启动市紧急救援中心、肝病防治中心和三级妇幼保健院建设。

　　继续开展文化建设年活动。加强精品创作，开展纪念辽沈战役胜利暨锦州解放70周年主题文化活动，举办群众文化节、全民读书节等文化惠民活动。加大文化遗产保护力度，加快文化产业示范基地建设，深化文化体制改革。筹建市博物馆新馆、少儿图书馆新馆，市文化艺术中心竣工投入使用。加强地方志、年鉴编修工作。

　　实施体育惠民工程。举办市民运动会等各类体育赛事，加快体育产业发展，不断满足人民群众多元化体育需求。

　　创建国家食品安全示范城市。深入实施食品药品安全整治三年行动计划和食品安全监管四年滚动计划，让全市人民吃的放心。

　　启动全国文明城市创建工作。大力促进民族团结、宗教和谐。全面提高双拥工作水平。

　　打造共建共治共享社会治理格局，推进平安锦州建设。完善立体化社会治安防控体系，实施派出所基础建设三年规划。推进“七五”普法，提升法律援助和人民调解工作水平。实施信访矛盾化解三年攻坚计划，减少信访存量，控制增量。严格落实安全生产责任制，强化灾害监测预警和应急管理体系建设，坚决防止较大以上安全事故，提升防灾减灾救灾能力。

　　（九）坚持加强政府自身建设，努力打造人民满意政府

　　新时代新任务，给政府工作提出了新标准新要求。我们要始终牢记打铁必须自身硬，坚持党的领导，扎实开展“不忘初心、牢记使命”主题教育活动，增强“四个意识”，坚决同以习近平同志为核心的党中央保持高度一致，切实把思想和行动统一到党的十九大精神上来，加快转变政府职能、提高行政效能，更好为全市人民服务。

　　坚持依法全面履职。严格依法行政，建设法治政府。加大政务公开力度。依法接受人大法律监督和工作监督，认真落实市人大及其常委会各项决议决定。自觉接受政协民主监督，积极支持市政协履行职能。主动接受社会和舆论监督。全面加强审计监督，突出抓好领导干部自然资源资产离任审计。认真办理人大议案、代表建议和政协提案。认真听取各民主党派、工商联、无党派人士和人民团体的意见建议。

　　持之以恒正风肃纪。坚持“一岗双责”，全面从严治党。始终把纪律挺在前面，严格执行中央八项规定精神，一以贯之反对“四风”，持续推进党风廉政建设和反腐败斗争。凡是群众反映强烈的问题都要严肃认真对待，凡是损害群众利益的行为都要坚决纠正，保证干部清正、政府清廉，大力营造海晏河清的良好政治生态。

　　勤勉尽责干事创业。锦州振兴发展的成就是全市干部群众干出来的，再创新业绩还要靠实干。我们要坚持干字当头、主动作为，动真碰硬、真抓实干，绝不纸上谈兵、绝不光说不练。强化督查和绩效考评，用足用好督考合一评价体系，严厉整肃庸政懒政怠政行为。健全正向激励机制和容错纠错机制，给干事者鼓劲，为担当者撑腰。提高狠抓落实本领，坚持说实话、谋实事、出实招、求实效，以钉钉子精神做实做细做好各项工作。

　　各位代表！我们已经迎来新的时代，踏上新的征程。梦想照亮前方，道路就在脚下，奋斗正当其时。让我们高举习近平新时代中国特色社会主义思想伟大旗帜，在省委、省政府和市委的坚强领导下，锐意进取，埋头苦干，为决胜全面建成小康社会、加快建设辽西区域中心城市而努力奋斗！
